# The effect of a commercial probiotic drink containing *Lactobacillus casei* strain Shirota on oral health in healthy dentate people

**DOI:** 10.3402/mehd.v24i0.21003

**Published:** 2013-10-29

**Authors:** Justyna Sutula, Lisa Ann Coulthwaite, Linda Valerie Thomas, Joanna Verran

**Affiliations:** 1 Microbiology Research Group, School of Health Care Science, Manchester Metropolitan University, Manchester, UK; 2 Yakult UK Ltd, Middlesex, UK

**Keywords:** Lactobacillus casei Shirota, transient colonization, LcS Select agar, oral microflora, saliva, tongue biofilm, Halimeter, OralChroma

## Abstract

**Background:**

In the past decade, the use of probiotic-containing products has been explored as a potential alternative in oral health therapy. A widely available probiotic drink, Yakult, was evaluated for oral health applications in this longitudinal study. Selected oral health parameters, such as levels and composition of salivary and tongue plaque microbiota and of malodorous gases, in dentate healthy individuals were investigated for changes. The persistence of the probiotic strain in the oral cavity was monitored throughout the study period.

**Methods:**

A three-phase study (7 weeks) was designed to investigate simultaneously the effect of 4-week consumption of the probiotic-containing milk drink Yakult on the microbiota of saliva and dorsum tongue coating in healthy dentate people (*n* = 22) and levels of volatile sulphur compounds (VSCs) in morning breath. Study phases comprised one baseline visit, at which ‘control’ levels of oral parameters were obtained prior to the probiotic product consumption; a 4-week period of daily consumption of one 65 ml bottle of Yakult, each bottle containing a minimum of 6.5×10^9^ viable cells of *Lactobacillus casei* strain Shirota (LcS); and a 2-week washout period. The microbial viability and composition of saliva and tongue dorsum coating were assessed using a range of solid media. The presence of LcS in the oral cavity was investigated using a novel selective medium, ‘LcS Select’. Portable sulphur monitors Halimeter^®^ and OralChroma^TM^ were used to measure levels of VSCs in morning breath.

**Results:**

Utilization of the LcS Select medium revealed a significant (*p* < 0.05) but temporary and consumption-dependent presence of LcS in saliva and tongue plaque samples from healthy dentate individuals (*n* = 19) during the probiotic intervention phase. LcS was undetectable with culture after 2 weeks of ceasing its consumption. Morning breath scores measured with Halimeter and OralChroma were not significantly affected throughout the trial, except in a small number of individual cases where Halimeter scores were significantly reduced during the probiotic intervention period. Natural fluctuations in resident acidogenic populations, and numbers of *Candida* and anaerobic species, including malodourous Gram-negative anaerobes, were unaffected.

**Conclusion:**

While no broad ecological changes in the mouth were induced by consumption of Yakult in healthy dentate individuals, findings of this study confirm the temporary and intake-dependent presence of LcS. Future studies could focus on subjects at greater risk of oral infection, where ill-defined microbiota (e.g. an increased presence of periopathogens) or clinically diagnosed halitosis might be significantly affected by consumption of this probiotic.

Probiotics are defined as live microorganisms which, when administered in adequate amounts, confer health benefits (1). Probiotic-containing products have long been studied and appreciated for their positive effect on gastrointestinal (GI) health. However, their beneficial role, supported by an increasing body of clinical evidence, reaches beyond the GI tract (2). In the past decade, studies on the potential applications of probiotic bacteria in the oral cavity for prevention and/or treatment of oral diseases have increased significantly (for reviews, see Refs. 3, 4).

It has been proposed that probiotic microorganisms may have anti-cariogenic activity by inhibition of mutans streptococci (5–7), and an oral malodour-reducing effect (8, 9). Application of probiotics in the treatment of gingivitis and periodontal disease (10, 11), as well as risk reduction of candidal mucosal infections in patients with denture-associated stomatitis, have also been described (12).

*Lactobacillus casei* strain Shirota (LcS), which is contained in the probiotic-fermented milk drink Yakult, has over 75 years’ history of safe consumption and proven health benefits, supported by extensive scientific research focused mainly on its reduction of functional and infectious gut diseases and its immune-modulating effect (13–16). There are many suggested mechanisms of the probiotic action of LcS in the intestine, but aside from immune modulation, the production of lactic acid (resulting in a reduction of local pH) and the competitive adhesion or displacement of pathogenic bacteria have been quoted most often in the literature (17, 18). Meurman (19) has proposed that in the oral cavity, probiotics may exhibit mechanisms similar to those seen in the gut, since the mouth is the first part of the GI tract. The mechanism of action of oral probiotics varies between species and can be multifactorial, but the main concepts include the mechanical displacement of pathogens and the production of organic acids and antimicrobial compounds. The application of Yakult for a beneficial effect on the oral cavity has overall received very little attention. Our *in vitro* studies have indicated the inhibitory properties of LcS on periopathogenic and malodorous species such as *Porphyromonas gingivalis* and *Fusobacterium nucleatum* (unpublished data). Four weeks of consumption of 6.5×10^9^ viable LcS per day by healthy denture wearers revealed a transient colonization of the oral cavity and denture surfaces by this strain during the consumption period and for up to 7 weeks of washout, as well as no significant effect on acidogenic populations such as levels of lactobacilli and streptococci (20).

This prospective study aimed to further investigate the transient nature of LcS in healthy dentate people during and post 4 weeks of consumption of Yakult. This investigation into the effect of Yakult consumption on balanced oral microbiota may provide a novel method for control of microbial populations of a pathogenic nature, such as those contributing to caries, periodontal disease, or halitosis. Salivary and tongue plaque microbial populations, including acidogenic and malodorous species, and the levels of malodourous gases in morning breath were monitored for changes.

## Materials and methods

### Study design

The study comprised three phases with a minimum of seven weekly visits to the laboratory and with samples of morning breath, saliva, and tongue coating being obtained weekly. Baseline levels of oral parameters were obtained during the first visit (participants acted as their own controls). This was followed by a 4-week intervention phase during which participants were consuming one 65 ml bottle of the probiotic-fermented milk drink Yakult per day after a meal (a total of 28 bottles). Subsequently, consumption of the study product was ceased (washout period), and the microbiological and breath sampling continued for a minimum of 2 weeks.

### Probiotic product

The study product, the fermented milk probiotic drink Yakult^®^ Light (supplied by Yakult UK Ltd), contains a single probiotic strain (LcS) at a minimum concentration of 6.5×10^9^ viable cells per 65 ml bottle. The viability tests performed in our laboratory on three randomly selected bottles from separate batches confirmed a high viable count of 1.2×10^10^ (±1.1×10^3^) cells per bottle. A supply of 28 bottles was provided for participants to store and consume at home. Participants were advised to keep their supply refrigerated, in order to preserve the viability of the probiotic bacteria. Instructions were given to drink the product after a meal (preferably at lunchtime and not right before bedtime), slowly taking small sips through a provided thin drinking straw (3 mm in diameter).

### Study group

A member of the research team assessed suitability for the study criteria. Suitable individuals who were willing to participate were provided with a participant information sheet, a consent form, leaflets describing probiotic usage and storage, and morning breath sampling instructions to retain. Informed consents were obtained on a separate occasion prior to participation. The study was approved by the Ethics Committee of the Faculty of Science and Engineering of the Manchester Metropolitan University. The trial was conducted according to the ethical principles of the Declaration of Helsinki (21).

Twenty-two healthy dentate volunteers working or studying at the Manchester Metropolitan University (14 males and 8 females, average ages 34 ± 11 years and 30 ± 12 years, respectively) who fitted the inclusion criteria were recruited. Participants were all self-proclaimed healthy non-smokers, without pre-existing oral irritations or systemic diseases. Participants were instructed not to change their normal oral procedures during the course of the study and to refrain from mechanical tongue scraping. During the probiotic intervention period, oral hygiene such as brushing teeth, rinsing the mouth, or flossing were to be postponed for at least 1 h after taking Yakult.

Exclusion criteria for the study were pregnancy, lactose intolerance or allergy to dairy products, a vegan diet (the product is made from skimmed cow's milk and therefore not suitable for vegans), smoking, oral antibiotics therapy within the last month or during the trial period, use of a chlorhexidine mouthwash within the last month, partial or full dentures, dental braces, pre-existing oral irritations or oral infections (rampant caries, severe gingivitis, advanced periodontitis, oral thrush, diagnosed halitosis, or diagnosed xerostomia), a history of intra-oral surgery within the last 6 months, poorly controlled diabetes, and systemic diseases.

### Sampling

Participants attended the Microbiology Research Laboratory once a week for a minimum of 7 weeks [one baseline visit; 4 weeks of probiotic intervention period visits (weeks 1, 2, 3, and 4); and 2 weeks of washout period visits (weeks 1 and 2)] for 30 min scheduled appointments between 8.00 and 10.00 am. On each test morning, the subjects were instructed to arrive without having performed any oral hygiene from midnight the night before. Subjects were asked to refrain from oral hygiene procedures and from consuming any food or drink for at least 12 h prior to visiting the laboratory and providing samples in order to increase breath levels. Drinking of water until 3 h before the examination was allowed. Subjects were asked not to wear strongly perfumed cosmetics on the morning of assessment. During these appointments, participants provided samples in the following sequence: malodour (‘morning breath’) assessment (mouth air sampling) using the Halimeter^®^ and OralChroma^TM^; collection of whole unstimulated saliva; and a tongue dorsum coating sample. The study did not include a placebo group. Subjects acted as their own controls (baseline period). As mentioned in the ‘Study group’ section, any oral hygiene procedures (brushing, rinsing, and flossing) were prohibited in the morning before sampling. Normal oral hygiene procedures were allowed throughout the study, with the exception of chlorhexidine-containing mouthwashes and any tongue-scraping practice, which were forbidden. Dietary restrictions included the consumption of medicated sweets and other probiotic-containing products (milk drinks, juices, tablets, and lozenges), and a maximum limit of four standard 120 g pots of yoghurt per week. Volunteers were also asked to avoid consumption of spicy and malodour-associated foods such as onions, garlic, chilli, and curry on the evening prior to the visit. Participants who were exposed to antibiotic treatment at any stage of the study were withdrawn from the trial and data analysis in order to avoid misrepresentation of the results.

### Assessment of morning breath odour with Halimeter and OralChroma

Morning breath samples were obtained using portable sulphide monitors: the Halimeter (Interscan Co., Chatsworth, CA, USA) and a portable gas chromatograph, OralChroma (CHM-1, Abilit, Osaka, Japan). The Halimeter was used for the detection and estimation of the peak value of total volatile sulphur compounds (VSCs) in parts per billion (ppb) (22). The OralChroma was used to measure the individual concentrations of hydrogen sulphide (H_2_S), methyl mercaptan (MM), and dimethyl sulphide (DMS) in ppb (23). Gas sampling was performed according to the manufacturer's instructions. Participants were supplied with a leaflet explaining the procedure, followed by demonstration of the sampling technique by one of the study investigators in preparation for participants’ self-sampling. Repeated measurements of mouth air collected with the Halimeter (three repeats) and OralChroma (two repeats) were averaged for each participant at weekly intervals. The order of air sampling was sequenced depending on the idle status of the OralChroma. An average gas analysis time for the OralChroma from injecting a sample is 8 min, followed by a 1-min interval standby mode time. Halimeter testing required a 3 min mouth air incubation period prior to sampling.

### Saliva and tongue plaque collection

Following completion of mouth air sampling, whole unstimulated saliva (approximately 3 ml) was collected by participants themselves by expectoration into a sterile plastic 25 ml bottle and kept on ice until processing within 2 h. Tongue plaque microbiota was sampled using a commercially available soft-bristle toothbrush on an approximately 2.16 cm^2^ sampling area, as previously described by Doran et al. (24). The sampling of tongue plaque was performed by subjects themselves by pressing and rocking the brush, which was positioned centrally on the dorsum region of the tongue (posterior third), five times. This method allows easier access towards the posterior part of the tongue, where-odour generating anaerobic flora can be found inhabiting tongue crypts at concentrations 10 times greater (up to 5×10^9^ cm^−2^) than in the front (25). Every week prior to sampling, participants were instructed to sample the same area of the tongue dorsum and apply the same amount of pressure, to ensure that each sample is as representative and reproducible as possible. Care was taken to avoid triggering the gag reflex.

### Quantitative microbiology

Saliva and tongue-coating samples were processed promptly within 2 h of collection. Collected saliva was serially diluted 10-fold to 10^−5^ dilution in sterile phosphate-buffered saline (PBS, pH 7.3; Oxoid, Basingstoke, UK). Immediately after collecting the tongue plaque, the toothbrush was returned to the boiling tube, and 10 ml of sterile reduced transport fluid (RTF), previously reduced overnight in an anaerobic cabinet, was transferred aseptically into the tube. The tube was vortex-mixed for 60 sec, and serial dilutions to 10^−3^ were prepared by adding 1 ml of tongue plaque suspended in RTF to 9 ml of PBS. The dilutions were prepared shortly before culturing to enhance the survival of fastidious species. Dilutions were plated onto a range of selective and indicator media (described in this section) using a spiral plater (Whitley Automatic Spiral Plater connected to Whitley Vacuum Source 602; Don Whitley Scientific Ltd, Shipley, UK). The remaining aliquots of each undiluted sample were divided into 1 ml aliquots and frozen without any processing within 4 h at −20°C for future investigation. Microbial counts were expressed in CFU ml^−1^ (saliva) and CFU cm^−2^ (sampled tongue dorsum).

Solid media used for culturing oral microorganisms included ‘LcS select’ agar, which was developed in our laboratory for selective and differential isolation of the probiotic strain LcS from a mixed culture (26). The formulation was based on de Man Rogosa Sharpe (MRS) agar medium with 1% vancomycin. The reducing agent L-cysteine HCl (Sigma-Aldrich, St. Louis, MO, USA) was added to LcS Select medium to improve the growth of lactobacilli under anaerobic conditions (27). Bromophenol blue (BPB) was used to indicate pH values from 3 to 5 that were generated by lactic acid bacteria, by production of a yellow colour at pH 3.0 and blue at pH 5. All plates were incubated in the presence of CO_2_ at 35°C for 72 h. Presumptive colonies of *L. casei* Shirota isolated were initially identified to genus based on a combination of colony morphology (round; convex or dome-shaped; 2–5 mm in diameter; with an entire margin; a smooth, shiny surface; an opaque, white or light-blue ring with a blue centre; and yellowing of the agar around the colonies, indicating acid production), Gram staining (Gram-positive average-sized or elongated non-spore-forming rods with rounded ends, often forming chains), and the results of a catalase test (negative).

Total facultative and obligate anaerobes were isolated on fastidious anaerobic agar (FAA; LabM, Bury, UK) supplemented with 5% defibrinated horse blood. Gram-negative anaerobic species, including black-pigmented anaerobes, were isolated on FAA that was additionally supplemented with G-N Anaerobe Supplement (GNA; Oxoid). Both FAA and GNA media were pre-reduced overnight before inoculation and incubated at 37°C in anaerobic conditions (80% N_2_, 10% H_2_, 10% CO_2_) for 5 days.

Aciduric bacteria (lactobacilli and other lactic acid bacteria) were isolated on MRS agar and incubated at 35°C in the presence of 5% CO_2_ for 24–48 h. Acid-producing organisms were explored using bromocresol purple indicator agar (BCP) (28) incubated at 35°C in the presence of 5% CO_2_ for 24 h. Periodically, colonies of interest were subcultured and identified to confirm the microbial group, and to give an indicator of the effectiveness of the selective media.

Sabouraud Dextrose agar (SABC; Oxoid) supplemented with chloramphenicol (LabM) was used for isolation of yeast, and it was incubated aerobically at 37°C for 3 days. Yeast isolates were tested for the production of true hyphae (*Candida albicans*) using the germ tube test (1 h 45 min incubation). Positive and negative control organisms were *C. albicans* GDH 2346 and *Candida krusei* NCYC 993, respectively. Additionally, Chromogenic Candida agar (Oxoid) was used for identification of *Candida* species. This medium, due to the presence of chromogenic substrates, allows differentiation of three *Candida* species by producing different-coloured colonies: *C. albicans* appears green, *C. krusei* produces purple-pink colonies, and *Candida tropicalis* appears blue. Control organisms were used: *C. albicans* GDH 2346, *C. krusei* NCYC 993, and *C. tropicalis* MMU1. Inoculated plates were incubated aerobically at 37°C for up to 72 h.

### Identification of LcS in oral samples

Enzyme-linked immunosorbent assay (ELISA) was performed on preserved samples of saliva and tongue collected during and after discontinuation of Yakult consumption to confirm the presence of LcS. Isolates confirmed by ELISA as LcS were further identified to the strain level using a molecular-sequencing method.

#### Identification of LcS isolates with ELISA using monoclonal antibodies

Frozen 1 ml aliquots of saliva and tongue plaque suspension collected during the intervention and washout periods were thawed, and a 10-fold dilution in PBS was cultured on LcS Select agar (26). A total of 20 presumptive LcS isolates were obtained at random for ELISA analysis.

LcS isolates and control organisms (LcS: positive control; and *L. casei* ATCC 4646 and *Lactobacillus rhamnosus* ATCC 7469: negative controls) were propagated in 10 ml volumes of sterile MRS broth (pH 6.2; Oxoid) in the presence of 5% CO_2_ for 48 h at 35°C. Turbid broths were pelleted at 3,000 rpm for 10 min at room temperature, and the cells were washed twice in sterile 0.05 M carbonate coating buffer (pH 9.6; ingredients per L: 15.9 g Na_2_CO_3_, 29.28 g NaHCO_3_, 2 g NaN_3_, and 1,000 ml double-distilled water). The optical density of the resuspended bacterial pellet was adjusted to 1.0 ± 0.1 at 660 nm and stored at 4°C overnight.

Qualitative analysis of the presumptive LcS isolates was performed by ELISA according to a method adapted from Yuki et al. (29). The dose–response relationship was determined using doubling dilutions of control antigen (LcS) to determine the minimum number of bacterial cells for detection and identification by the assay. Controls used were positive (50 µl of antigen) and negative (50 µl of carbonate buffer).

Briefly, 100 µl of an appropriate concentration of antigen (LcS isolates) or control antigen (LcS, *L. casei* ATCC 4646, or *L. rhamnosus* ATCC 7469) diluted in 0.05 M carbonate buffer 1:10 and 1:20 was added to wells in a 96-well, flat-bottom, high-affinity, protein-binding ELISA plate (Nunc™ Maxisorp™, eBioscience, San Diego, CA, USA). The plate was covered in adhesive plastic to prevent evaporation and incubated at 4°C overnight.

The excess of antigen was discarded, and the plate was dried by tapping it upside down on dry paper towels. Wells were washed with wash buffer (200 µl/ well; PBS supplemented with 1% Triton-X 100).

Non-specific protein bindings were blocked by adding 200 µl per well of blocking buffer containing 1% bovine serum albumin (BSA) solution in 0.05 M carbonate coating buffer, and incubating at 37°C for 1 h. The wash step was repeated three times. Washed wells were treated with 100 µl per well of the primary monoclonal antibody L8 (provided by Yakult Central Institute for Microbiological Research, Tokyo, Japan) diluted 1:20 in 1% BSA carbonate buffer and incubated at 37°C for 1.5 h.

The wash step was repeated three times. Anti-mouse IgM peroxidase-labelled secondary antibody (Sigma-Aldrich) diluted 1:1,000 in 1% BSA carbonate buffer was added to wells (100 µl/well) and incubated at 37°C for 1.5 h, and wells were washed three times. The substrate solution (0.4 mg/ml oPD 1,2-phenylenediamine dissolved in 0.05 M phosphate–citrate buffer, containing 0.03% sodium perborate and 0.02% of 30% H_2_O_2_ (added immediately before use); pH 5.0) was added (200 µl/well), and the plate incubated at 37°C for 10 min. The reaction was stopped with 50 µl per well of 2.5 M sulphuric acid, and the absorbance was measured at 490 nm.

A positive reaction (i.e. the presence of LcS) was indicated by a change in colour of the solution to yellow. LcS-negative samples remained colourless. A range of absorbance values was given for the positive control (LcS). Absorbance values of tested isolates were classified as positive (indicative of LcS presence) if they fell into the positive range determined from the dose–response relationship. Values below or above the ‘LcS range’ were identified as negative (no LcS present).

#### Molecular identification of LcS-positive isolates detected with ELISA

The DNA from LcS-positive isolates detected with LcS-specific antibodies using ELISA, and control species of lactobacilli (LcS, *Lactobacillus paracasei* F19, and *Lactobacillus fermentum* NCIMB6991), was extracted using a DNeasy Blood and Tissue kit (Qiagen, Valencia, CA, USA) according to the manufacturer's instructions. Extracted DNA samples were stored in microcentrifuge tubes at −20°C until required. The purity of the extracted DNA fragments was confirmed by horizontal gel electrophoresis.

PCR amplification was performed on LcS-positive isolates and controls (*n* = 14). The reaction mixture contained (volumes for 10 reactions) 1.0 µl universal 518R reverse primer (5’-GTA TTA CCG CGG CTG CTG G-3’), 1.0 µl universal 101F-GC clamp forward primer (5’-CGC CCG CCG CGC CCC GCG CCC GTC CCG CCG CCC CCG CCC GTC GCG GAC GGG TGA GTA A-3’), 25 µl 10×NH_4_ buffer, 7.5 µl MgCl_2_, 0.5 µl dNTPs, 215 µl molecular water, and 0.5 µl *Taq* polymerase. Each reaction tube contained 24 µl of the master mix and 1 µl of extracted DNA sample (isolates and control lactobacilli). Control organisms were LcS, *L. paracasei* F19, and *Lactobacillus plantarum* NCIMB 8014. The negative control consisted of master mix and target DNA replaced with molecular water. The amplification program comprised one cycle of 95°C for 5 min, 30 cycles of 94°C for 30 sec, 30 cycles of 50°C for 20 sec, 30 cycles of 60°C for 4 min, and one cycle of 72°C for 3 min. Following the amplification process, the primers, nucleotides, polymerases, and salts were removed from amplified DNA fragments using the QIAquick PCR Purification kit (Qiagen) according to the manufacturer's protocol. Purified PCR products were sequenced using a BigDye™ Terminator Cycle Sequencing Ready Reaction Kit (Applied Biosystems, Carlsbad, CA, USA) and an automatic sequencer (model 3730; Applied Biosystems) at the DNA Sequencing Facility at the University of Manchester. The sequences were tailored using freeware Chromas Lite version 2.01. Analysis of 16S rRNA sequences was conducted using GenBank via the database sequence search engine Basic Local Alignment Search Tool (BLAST; http://blast.ncbi.nlm.nih.gov/Blast.cgi). Identities of sequences were determined on the basis of the highest score of similarity.

### Analysis

A one-way ANOVA test was used to investigate the effects (if any) of the probiotic consumption on microbial numbers in saliva and tongue coating, and levels of VSCs in mouth air. A value of *p* < 0.05 was considered significant. The Tukey 95% simultaneous confidence intervals test was used to perform a pairwise comparison of VSC readings to establish groups with significant difference, once ANOVA returned a *p* value of < 0.05. Pearson correlation was used to determine any associations between concentrations of VSCs determined by the Halimeter and OralChroma, VSCs and viable counts, and viable counts of salivary and tongue plaque microorganisms. All statistical analysis was performed using the statistical software MINITAB^®^ version 15.

## Results

All recruited subjects completed the study except for one female volunteer, who was prescribed a course of antibiotics during participation and therefore was withdrawn from the trial and data analysis (resulting sample size *n* = 21). There was considerable variation in the data of all parameters measured.

### Morning mouth air odour analysis

**Fig. 1:**
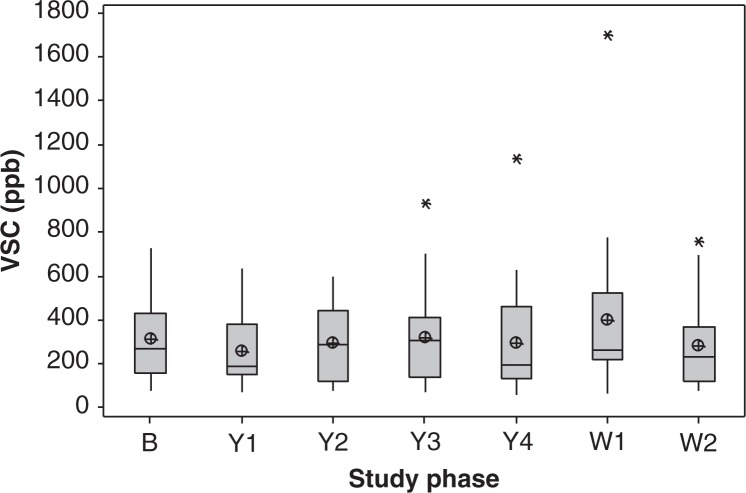
Boxplot of the averaged concentrations of total volatile sulphur compounds (VSCs) detected in the mouth air of healthy dentate individuals (*n* = 21) using the Halimeter^®^ at weekly intervals (three repeats per measurement): at baseline prior to the probiotic intervention period (B), at 1–4 weeks of probiotic consumption (Y1–Y4), and at 1–2 weeks of the washout period (W1–W2). No significant difference between averaged weekly scores was found (*p* = 0.593). The median is represented by the horizontal line. The mean value is represented by a crossed dot. Asterisks represent outliers.

There was no significant difference in average concentrations of total VSCs measured with the Halimeter between study phases (*n* = 21, *p* = 0.593; Fig. 1). Large variations in inter- and intra-individual measurements were observed. Six individuals had consistently lower average scores (<200 ppb; nos. 1, 4, 5, 9, 13, and 14) throughout the study. The remaining individuals presented largely variable weekly scores, and no trends were found. There was a significant decrease (24–34%) in VSCs during the Yakult phase in comparison to baseline (*p*<0.05) for four participants. Conversely, a significant increase (*p* ≤ 0.034) in average VSCs was observed in three participants during the intervention period.

**Fig. 2:**
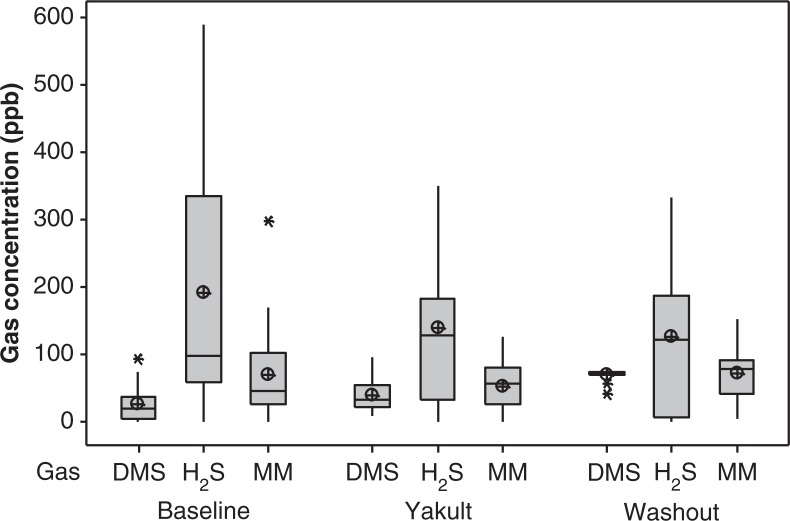
Boxplot of averaged concentrations in parts per billion (ppb) of dimethyl sulphide (DMS), hydrogen sulphide (H_2_S), and methyl mercaptan (MM) detected in the mouth air of healthy dentate individuals (*n* = 21) using the OralChroma^TM^ prior to the probiotic intervention period (Baseline), during the 4-week Yakult consumption phase (Yakult; averaged weekly readings), and for 2 weeks post-intervention (Washout; averaged weekly readings). The median is represented by the horizontal line. The mean value is represented by a crossed dot. Asterisks represent outliers.

An apparent decrease in average concentrations of hydrogen sulphide (H_2_S) and MM, measured with the OralChroma, was observed during the Yakult phase in comparison to baseline (Fig. 2), but this was not significant (*p*=0.292 and *p*=0.446, respectively). Following discontinuation of the probiotic consumption, concentrations of H_2_S were again reduced, but not significantly so. Average concentrations of MM at washout returned to baseline levels. There was a significant increase in DMS during the washout period (*p*=0.001), when compared to baseline and Yakult average scores (Fig. 2 and Table 1).

**Table 1:** Averaged concentrations in parts per billion (ppb) of dimethyl sulphide (± standard deviation) detected in the mouth air of healthy dentate individuals (*n* = 21) using the OralChroma^TM^ prior to the probiotic intervention period (Baseline), during the 4-week Yakult consumption phase (Y; readings taken at weekly intervals), and for 2 weeks post-intervention (Washout; readings taken at weekly intervals)

DMS (ppb)	Baseline	Y1	Y2	Y3	Y4	W1	W2
Mean	27.1	25.6	28.5	40.9	55.4	71.1	75.4
	(±25.8)	(±19.9)	(±37.0)	(±29.4)	(±37.3)	(±37.4)	(±29.9)
Median	20.5	21.5	19.0	35.5	46.0	68.0	80.8

### Microbiological analysis

**Fig. 3:**
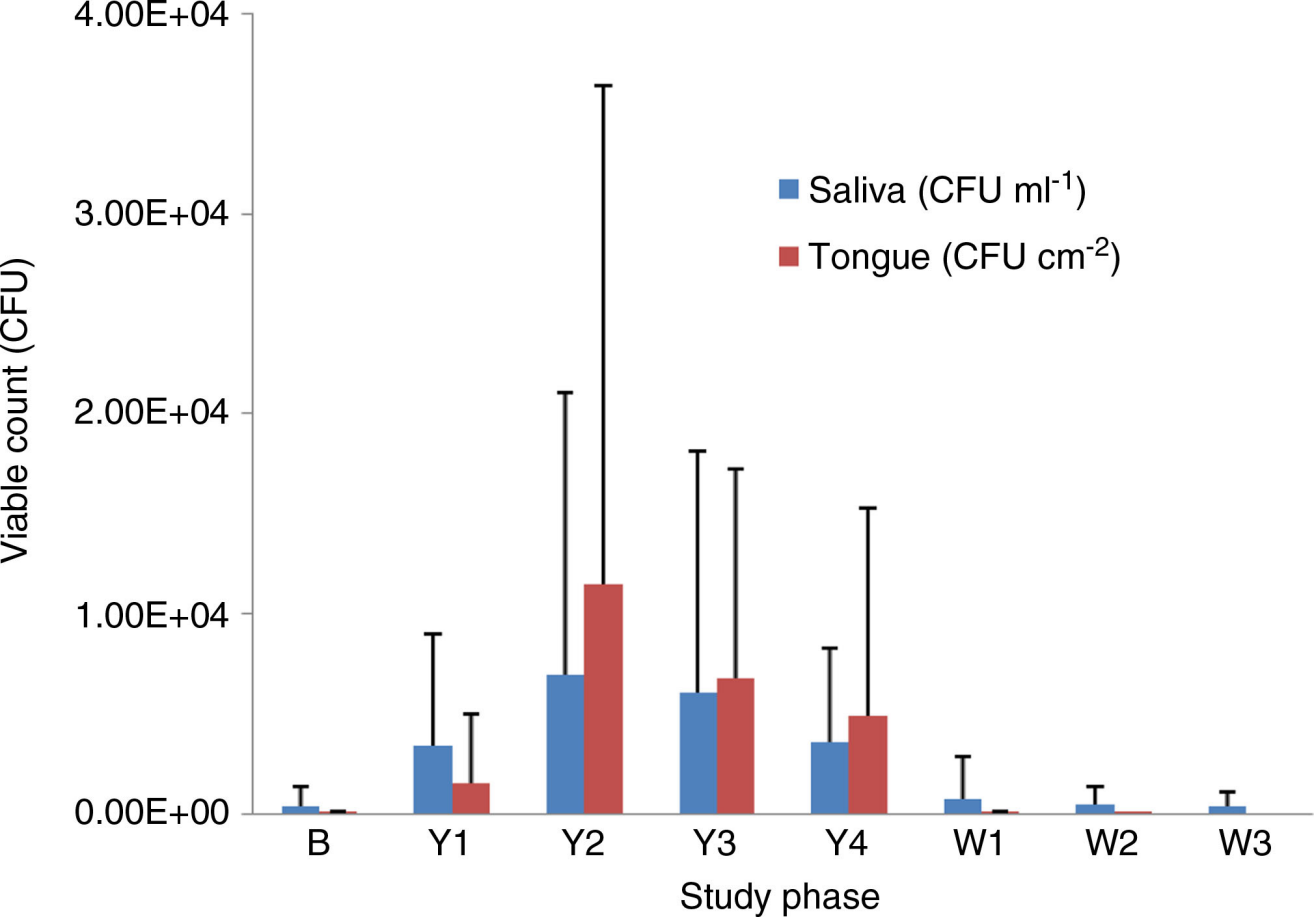
Averaged count of colony-forming units of *Lactobacillus casei* Shirota isolated on LcS Select medium from saliva (CFU ml^−1^) and tongue plaque (CFU cm^−2^) obtained from 21 participants at weekly intervals: baseline (B), weeks 1–4 of the probiotic intervention period (Y1–Y4), and weeks 1–3 of the washout period (W1–W3). Error bars represent standard deviation.

The average viable count of LcS, as determined using LcS Select medium, was significantly increased in the saliva during the probiotic intervention period when compared with the baseline (weeks 1, 3, and 4; *p*<0.05; Table 2 and Fig. 3). During the discontinuation phase, the viable count reduced significantly when compared with the probiotic phase (*p*<0.05).

**Table 2:** Summary of the averaged viable counts (± standard deviation) of bacteria and yeast isolated from saliva (CFU ml^−1^) and tongue biofilm (CFU cm^−2^) of healthy dentate individuals (*n* = 21) on a range of media at baseline, during the 4-week Yakult consumption phase (Yakult; averaged weekly counts), and for 2 weeks post-intervention (Washout; averaged weekly counts)

	Baseline	Yakult	% Difference	Washout	% Difference
Saliva (CFU ml^−1^)					
MRS	5.3×10^6^ (±7.4×10^6^)	2.8×10^6^ (±4.5x 10^6^)	−0.5	2.3×10^6^ (±3.4×10^6^)	−0.6
LcS Select	3.7×10^2^ (±9.6×10^2^)	5.0×10^3^ (±9.9×10^3^)	12.6†	4.4×10^2^ (±8.4×10^2^)	0.2
FAA	2.3×10^8^ (±2.8×10^8^)	1.9×10^8^ (±2.2×10^8^)	−0.2	2.4×10^8^ (±2.5×10^8^)	0.1
GNA	6.7×10^7^ (±1.2×10^8^)	3.2×10^7^ (±6.4×10^7^)	−0.5	6.3x 10^7^ (±1.3×10^8^)	−0.1
Black (GNA)	9.6×10^5^ (±2.5×10^6^)	8.3×10^5^ (±2.3×10^6^)	−0.1	1.0×10^6^ (±2.2×10^6^)	0.1
SABC*	6.6×10^2^ (±1.7×10^3^)	2.0×10^3^ (±1.3×10^3^)	2.0	3.5×10^3^ (±1.4×10^4^)	4.4
BCP (% acid)	28.6 (±22.4)	32.9 (±24.9)	0.1	41.5 (±28.1)	0.4
Tongue (CFU cm^−2^)					
MRS	3.7×10^6^ (±7.8×10^6^)	1.4×10^6^ (±2.3×10^6^)	−0.6	1.0×10^6^ (±2.1×10^6^)	−0.7
LcS Select	2.1×10^1^ (±9.9×10^1^)	6.1×10^3^ (±1.5×10^4^)	291.0†	1.9×10^1^ (±8.4×10^1^)	−0.1
FAA	2.8×10^7^ (±2.7×10^7^)	2.8×10^7^ (±3.1×10^7^)	0.0	3.5×10^7^ (±5.3×10^7^)	0.2
GNA	1.1×10^7^ (±9.6×10^6^)	8.1×10^6^ (±9.5×10^6^)	−0.3	9.0×10^6^ (±1.3×10^7^)	−0.2
Black (GNA)	3.0×10^5^ (±6.3×10^5^)	1.8×10^5^ (±5.0×10^5^)	−0.4	1.1×10^5^ (±2.3×10^5^)	−0.6
SABC*	2.1×10^2^ (±9.9×10^2^)	3.2×10^2^ (±2.0×10^2^)	0.5	2.4×10^2^ (±8.5×10^1^)	0.2
BCP (% acid)	40.6 (±22.1)	49.2 (±26.1)	0.2	53.4 (±26.7)	0.3

Differences between the averaged colony counts at Yakult and Washout are expressed as a percentage of the baseline count. Positive values indicate an increase in CFU, and negative values a decrease.

Key: Media used for isolation of bacteria and yeast: MRS (lactic acid bacteria); LcS Select (*L. casei* Shirota); FAA (total facultative and obligate anaerobic species); GNA (obligate anaerobic species); Black GNA (black-pigmented obligate anaerobic species); SABC (yeast); BCP (acidogenic bacteria expressed as a percentage of acid-producing bacteria)

* *n* = 8 yeast carriers

† significance of paired *t-*test comparing averaged CFU counts at Baseline and Yakult or Baseline and Washout (*p* < 0.05).

Two weeks after the probiotic discontinuation, colonies preliminarily identified as LcS were present in the saliva of 11 participants (9 male and 2 female). Those participants were recalled at 3 weeks of washout to provide additional saliva samples, which were screened for LcS, yeast, and acidogenic microorganisms. Seven participants agreed to repeat the sampling. In five, LcS was isolated from the saliva at lower concentrations in comparison to the second week of washout (on average, a 70% reduction in the viable count). No LcS was isolated from the saliva of the remaining two participants. In eight individuals, colonies morphologically resembling LcS were isolated from saliva at baseline. Those numbers either increased during the probiotic consumption phase and returned back to baseline levels at washout, or did not change throughout the trial.

Presumptive LcS colonies were isolated from the tongue dorsum plaque of 19 out of 21 individuals up to a concentration of 9.4×10^4^ CFU cm^−2^ during the probiotic consumption period. There was a significant increase in the viable count of LcS in tongue plaque during the probiotic consumption period compared with baseline levels (*p* < 0.05; Table 2). At baseline, no LcS was detected in the tongue plaque of any participants except for one individual (no. 22). Following 2 weeks of washout, no LcS was present in the tongue coating of any individuals with the exception of one at a concentration of 2.3×10^2^ CFU cm^−2^ (no. 21). No LcS was detected at any time in either the saliva or tongue plaque of one volunteer (no. 16). All presumptive LcS colonies isolated on LcS Select agar were round, convex, and 2–5 mm in diameter, with an entire margin; a smooth, shiny surface; and an opaque, light-blue colour (or a light-blue ring and dark-blue centre), with yellowing of the agar around them (due to acid production). Gram stain confirmed the characteristics of lactobacilli: Gram-positive average-sized or elongated rods with rounded ends, often forming chains. The catalase test was negative for all isolates.

Overall, there were no significant changes in microbial counts on other media. At baseline, 8 out of 21 participants carried yeast in saliva (38%) but not on the tongue. Within this group, there was no significant difference in yeast count throughout the trial (Table 2). Identification of the species revealed 48% prevalence of *C. albicans* (10 out of 21 isolates). Three participants carried more than one *Candida* species, including *C. albicans*, *C. parapsilosis*, *C. guilliermondii*, or *C. krusei*. At the third week of washout, there was no difference in yeast count in the saliva of participants still carrying LcS. In one individual (no. 11), yeast was recovered from saliva after 2 weeks of taking the probiotic-containing product and remained until the end of the trial at a low concentration. In one individual (no. 14), yeast was isolated from both the saliva and tongue dorsum throughout the study, including baseline, but consumption of the probiotic-containing product had no effect on the viable count.

The viability of salivary and tongue plaque lactic acid bacteria estimated on MRS agar did not change significantly during and after the intervention period when compared with the baseline levels (Table 2). There was a non-significant increase in the proportion of acid-producing microorganisms isolated on BCP during the Yakult phase and extended washout when compared to baseline for both saliva and tongue plaque (Table 2).

The total number of facultative and obligate anaerobic species did not change significantly during the study. A non-significant decrease in Gram-negative obligate anaerobic bacteria by over 50% in saliva and 25% in tongue plaque was observed at the time of the probiotic intervention phase. The numbers of black-pigmented species were also reduced when compared to baseline, but with no significant difference. After discontinuation of the probiotic consumption, the viability of black-pigmented species increased in the saliva to baseline level, but it remained reduced on the tongue for up to 2 weeks (Table 2).

#### ELISA and molecular identification results

Of 20 randomly selected LcS-resembling isolates previously recovered from frozen samples of saliva and tongue biofilm using the LcS Select medium, 11 reacted with the monoclonal L8-antibody (55%; average OD_490nm_ 0.122 ± 0.034), based on duplicate samples tested by ELISA on two different occasions. The positive and negative controls produced average absorbance values of OD_490nm_ 0.105 (± 0.049) and 0.085 (± 0.027), respectively, coupled with colour indication (increase in absorbance value and yellow colour when positive). Out of the positive results, 10 isolates originated from the tongue plaque, which were collected at 2 and 4 weeks of Yakult consumption. In one subject (no. 17), LcS was present in the saliva after 3 weeks of washout.

Sequence analysis of 10 isolates identified as LcS using ELISA and LcS-specific antibodies showed significant similarities (≥ 99%) to *L. paracasei* (*n*= 6, accession no. GU425013.1) and *Lactobacillus paracasei* subspecies *paracasei* (*n* = 4, accession no. HQ697655.1) when compared to the deposited sequences using the BLAST sequence search engine. Most identical matches to control species LcS, *L. paracasei* F19, and *L. plantarum* NCIMB 8014 were *L. paracase*i subspecies *paracasei* (accession no. AB300210.1), *L. paracasei* (accession no. AB368899.1), and *L. plantarum* (accession no. HQ697655.1), respectively.

## Discussion

Probiotic microorganisms such as some *Lactobacillus* spp. have been described as promoting a healthy digestive system and immunomodulation. There is reasonable evidence that some probiotic species may also have a beneficial effect on oral health (30, 31). LcS is one of the most studied probiotic strains with scientifically proven health benefits (32). Despite the amount of research relating to its effects on GI health, the effect of Yakult consumption on oral health parameters has received limited attention (11, 33).

Recently, we demonstrated the transient and consumption-dependent nature of this probiotic bacterium in a group of complete denture wearers following a 4-week period of Yakult consumption. LcS was detected by culture in saliva and in tongue and denture biofilm up to 7 weeks from ceasing consumption, but there was no indication of permanent colonization (20).

In this study, the effect of 4-week consumption of Yakult containing LcS on oral microbiota in healthy individuals with full dentition was evaluated. For the first time, the effect of Yakult consumption on the levels of VSCs present in morning breath was investigated. The capability of LcS to colonize oral surfaces and persist during and post-consumption in saliva and tongue biofilm was also investigated using selective culturing and molecular identification methods.

The modified medium LcS Select used in this study proved useful for differential isolation and quantification of presumptive LcS from saliva and tongue plaque. Fifty-five percent of tested salivary and tongue plaque isolates were positive for the presence of LcS-specific antigen detected with ELISA, and a marked ≥ 99% similarity of amplified sequences to *L. paracasei* and *L. paracasei* subspecies *paracasei*. The probiotic strain LcS belongs to the species *L. paracasei* according to recent reclassification (34). Utilization of LcS Select medium revealed a presence of LcS in saliva and colonization of the dorsum region of the tongue (up to 10^3^ CFU per millilitre of saliva and per square centimetre of tongue surface) during Yakult consumption. During the washout period, counts decreased to pre-treatment levels, indicating that this strain did not colonize the oral surfaces permanently. These findings are in agreement with the results of our previous investigational study involving complete denture wearers, where LcS was detected in saliva, in dorsal tongue plaque, and on denture surface during and post-intervention, but for a limited time only (20).

The ability of probiotics to colonize oral surfaces during intervention has received some attention (10, 35–38). Colonization is thought to be required in order to alter the composition of established biofilm, such as subgingival or tongue plaque. The authors (10, 35–38) found that probiotics were only transiently present in oral samples (mainly saliva) during the consumption period and shortly after discontinuation of the treatment, eventually becoming undetectable. In terms of permanent colonization of the oral cavity, there is only one case of stable and long-term establishment of probiotic described by Yli-Knuuttila et al. (37), in which an individual received *Lactobacillus rhamnosus* strain GG probiotic therapy at the age of 10 years and became permanently colonized. The mechanism of this phenomenon is unknown; however, some speculate that the resident microbiota in children is still immature and thus easier to modify (39). The evidence from intestinal studies also confirms a transient and consumption-dependent character of LcS (13). It is known that LcS can be detected in faecal samples for only a limited period after the end of probiotic ingestion (29).

Despite the differences in dose, frequency (once to a couple times per day), or duration of probiotic consumption (mainly a couple of weeks) described in *in vivo* trials, no permanent colonization of oral surfaces has been reported. Authors have also investigated a variety of delivery vehicles such as liquid (milk, yoghurt, juice, or mouthwash), solid (cheese, lozenge, tablet, or capsule), and various other devices (chewing gum, straws containing probiotic, or pacemakers) in order to investigate whether increased contact time with oral surfaces improves colonization. Consequently, it has been proposed that, regardless of the vehicle, in the presence of the indigenous microbiota found in adults, repeated application of dietary probiotics is necessary to maintain the desired probiotic effect. It is possible that the already established microbial ecology prevents introduced probiotics from colonization and becoming a part of the commensal biofilm.

There were no clear indications as to whether the presence of LcS affected existing microbial populations in the mouth. Consumption of LcS did not appear to have any effect on naturally occurring oral candida found in 38% of participants, regardless of whether it was a constant or temporary carriage of the yeast. Consumption-dependent colonization of LcS had no significant effect on the viable count of salivary or tongue plaque candida throughout the study. According to our *in vitro* inhibition studies (unpublished data), neither LcS nor Yakult exerted antifungal properties on monocultures of type strains of *Candida* or clinical candida isolates (*C. albicans*, *C. krusei*, *C. tropicalis*, and *C. guilliermondii*).

Measuring naturally elevated concentrations of VSCs found in the morning breath is generally recognized as a surrogate method for evaluating the effectiveness of various halitosis treatments (40). With the exception of some individual cases, there was no overall significant effect on morning breath measured with the Halimeter and OralChroma during the Yakult phase. This may be due to the large variation between repeated readings in the majority of subjects. While the present study was carried out with a limited number of individuals, it is worth noting that a pilot study performed in our laboratory, which involved 19 healthy participants and the same Yakult-drinking regime, showed very similar Halimeter results to this trial, which strengthens the outcome of the current investigation (results not presented).

In this study, a portable gas chromatograph OralChroma (23) was used to measure individual concentrations of the three gases in morning mouth air. In our study, H_2_S and MM were present in by far the highest concentrations, and this agrees with the literature (41). It was found that during the probiotic intervention and washout periods, average concentrations of H_2_S and MM decreased in relation to baseline readings. The concentration of DMS was much lower in contrast to other two gases. Interestingly, the concentrations of DMS significantly increased during the Yakult and washout phases. An increased level of DMS in breath has been associated with the extra-oral bloodborne odour, estimated to comprise around 10–15% of halitosis (42). Although probiotic bacteria are capable of generating considerable levels of VSCs, including H_2_S and DMS (43), this increase is unlikely to be associated with intestinal probiotic digestion. However, the presence of DMS in breath is of extra-oral origin (or bloodborne) and can be detected in exhaled mouth and nose breath according to Tangerman and Winkel (42). Molecules of internally generated DMS can be absorbed into the bloodstream and later transferred into the alveolar air and be expired. Excretion of these volatiles then causes bad breath, which is detectable in mouth as well as nose breath (44). In this study, we measured only air collected in the mouth (not mouth breath) to determine the concentration of malodorous gases produced by resident oral bacteria. Thus, we could not confirm whether or not the increase of DMS was a result of the ingested probiotic strain producing VSCs; reasons remain to be elucidated.

Gram-negative anaerobic species associated with malodour can be inhibited by the low pH produced by acidogenic members of the oral microbiota (45, 46). An increase in the acidogenic population on the tongue may result in a decrease in oral malodour – although the substrate for acid production (prebiotic) and the acid producers (probiotic) must not, of course, be cariogenic. In our study, we report a non-significant reduction of malodour-forming bacteria, including black-pigmented species, after Yakult consumption. Additionally, a significant negative correlation between an increased acidogenic ratio and reduced Halimeter scores was observed (results not presented). This may suggest that the presence of LcS and acidogenic populations during the Yakult phase reduces the viability of obligate anaerobic species and subsequently the formation of VSCs detected with the Halimeter. It has also been found that LcS can inhibit periodontopathogens such as *Porphyromonas gingivialis* and *Fusobacterium nucleatum* *in vitro* using a well-diffusion method. No clinical parameters of periodontal health such as probing depth or bleeding on probing were measured in this study.

In a healthy, well-balanced oral microbiota, no significant changes in parameters would be desirable; however, the results provide useful insight into the effects of Yakult consumption in a healthy mouth. Trends observed indicate that no broad ecological changes in the mouth were induced by consumption, with temporary colonization by the probiotic LcS and potentially desirable shifts in other populations such as reduction in black-pigmented anaerobic species being observed.

## Conclusion

In conclusion, 4-week consumption of one bottle per day of Yakult containing a minimum of 6.5×10^9^ viable cells of probiotic *L. casei* strain Shirota per 65 ml bottle had no significant effect on oral parameters in healthy dentate participants. Overall, microbial fluctuations in saliva and tongue plaque, and the morning mouth air scores, were not affected during the intervention period, except in individual cases. There was, however, a large variability in microbial counts and breath results within and between participants. The modified medium LcS Select enabled differential quantification of LcS from saliva and tongue plaque samples. The probiotic strain was detected in the oral cavity during and 2 weeks after consumption, demonstrating transient colonization. Future studies could focus on subjects at greater risk of oral infection, where ill-defined flora, for example an increased presence of periopathogens or clinically diagnosed halitosis, might be significantly affected by consumption of this probiotic.
